# Resveratrol reverses Doxorubicin resistance by inhibiting epithelial-mesenchymal transition (EMT) through modulating PTEN/Akt signaling pathway in gastric cancer

**DOI:** 10.1186/s13046-016-0487-8

**Published:** 2017-01-26

**Authors:** Jiahui Xu, Deying Liu, Huilin Niu, Guifang Zhu, Yangwei Xu, Danli Ye, Jian Li, Qingling Zhang

**Affiliations:** 1 Nanfang Hospital/First Clinical Medical School, Southern Medical University, Guangzhou, 510515 China; 2 Department of Pathology, School of Basic Medical Sciences, Southern Medical University, Guangzhou, 510515 China; 3 Department of Pathology, Nanfang Hospital, Southern Medical University, Guangzhou, 510515 China

**Keywords:** Resveratrol, Doxorubicin, Drug resistance, EMT, PTEN/Akt

## Abstract

**Background:**

Gastric cancer is one of the major causes of cancer-related mortality worldwide. Most of patients presenting with inoperable gastric cancers rely on systemic chemotherapy for prolongation of survival. Doxorubicin (DOX) is one of the important agents against gastric cancer. Acquired DOX-resistance severely impedes the chemotherapeutic effect, invariably leading to poor prognosis. Resveratrol (RES) as a kind of phytoalexin has demonstrated anti-tumor functions in breast cancer and myeloid leukemia, but its function and mechanism are still unknown in gastric cancer treatment.

**Methods:**

CCK8 assay was used to detect the cytotoxicity of DOX and RES to gastric cancer cells. DOX-resistant subclone cell line (SGC7901/DOX) was derived from SGC7901 cells exposed to stepwise increasing concentrations of DOX treatment. We measured the migratory capabilities of SGC7901/DOX cells by Cell scratch test and Transwell assay. SGC7901/DOX cells were treated with DOX, RES, neither or both. Then we analyzed cell survival by CCK8 assay, colony formation by Colony-forming assay, cell apoptosis by Annexin-V-FITC and PI dual staining assay and cell migration by Cell scratch test and Transwell assay. Western blotting was conducted to detect the protein expressions of PTEN/Akt signaling pathway and EMT-related markers. Immunofluorescence was performed to confirm the EMT-related markers expressions. The xenograft model was used to assess the effect of DOX and RES in vivo. The key molecules associated with proliferation, apoptosis and EMT were evaluated by immunohistochemistry in tumor specimens.

**Results:**

SGC7901/DOX cells acquired drug resistance and enhancive migratory capability. RES enabled SGC7901/DOX cells to regain DOX sensitivity, mitigated the aggressive biological features, promoted cell apoptosis in vitro and inhibited tumor growth in vivo. Mechanistic studies revealed that SGC7901/DOX cells underwent epithelial-mesenchymal transition (EMT) which was induced by Akt activation, and through activating PTEN, RES inhibited the Akt pathway, and then achieved the reversion of EMT.

**Conclusion:**

RES serves as a novel solution to reverse the DOX-resistance of gastric cancer via preventing EMT by modulating PTEN/Akt signaling pathway. DOX-RES combined treatment provides a promising future for gastric cancer patients to postpone drug resistance and prolong survival.

## Background

Gastric cancer is the fourth common malignancy in males and the fifth in females. It accounts for about 9% of worldwide cancer deaths in 2012 [1]. Most newly diagnosed cases present with locally advanced or metastatic gastric cancers [2, 3] Still those patients with operable gastric adenocarcinoma would have a high risk of recurrence after tumor complete resection despite improvements in the surgical treatment [4]. Chemotherapy has long been considered as a sophisticated treatment of locally advanced and metastatic gastric cancers [5, 6]. Doxorubicin (DOX), an anthracycline-based anti-tumor drug combined with other chemotherapeutic agents including fluorouracil and mitomycin, was first introduced as a gold therapy regimen for advanced gastric cancer patients in 1980 [7]. However, some clinical trials reported traditional DOX-based regimens failed to prolong survival and brought about some adverse effects for gastric cancer patients [8–11]. Recent in vitro study indicated that the frequent acquisition of DOX resistance and the potential induction of epithelial–mesenchymal transition (EMT) might be crucial in the limitation of DOX treatment in gastric cancer [12]. To find out a novel solution to alleviate the adverse effects in an all-round way, we endeavor to discover a chemo-sensitizer and side-effects attenuator for DOX in gastric cancer chemotherapy as well as its molecular mechanism.

A number of phytochemicals available exhibit chemo-preventive effect and sensitize cancer cells toward DOX [13]. Among various phytochemicals, resveratrol (RES), a self-protective polyphenolic phytoalexin derived from plants, has attracted increasing attention due to its multiple health benefits [14]. In addition to the pharmacological effects of RES on the aging process, diabetes, neurological dysfunction, cardiovascular system and inflammation, its potent anti-tumor activity were closely observed in studies including breast cancer, lung cancer, prostate cancer, hepatocaracinoma, colorectal cancer and so on [14–16]. The combined treatment of DOX and RES achieves a synergistic effect and reverses multidrug-resistance in breast cancer cells [17] and acute myeloid cells [18]. As DOX is one of the key chemotherapy agents in gastric cancer treatment, the combination of DOX and RES turns out to be a potentially promising chemotherapy regimen for gastric cancer and the relevant mechanism remains to be explored.

In this study, DOX-resistant human gastric cancer cell line (SGC7901/DOX) was established and demonstrated aggressive migratory capabilities. Then RES was introduced and it successfully alleviated cell migration and chemo-sensitized DOX through reversing EMT process and regulating PTEN and Akt/mTOR pathway. Our work sheds new light on RES as a potential adjuvant to DOX in gastric cancer treatment to solve drug resistance and tumor metastasis. And the relevant mechanism study and prospective clinical trials are expected.

## Methods

### Cell culture

Human gastric cancer cells SGC7901 and MGC803 were obtained from American Type Culture Collection Cell Biology Collection (ATCC, Manassas, VA, USA) and maintained in Department of Pathology, Southern Medical University (Guangzhou, China). Cells were cultured in RPMI 1640 medium supplemented with 10% fetal bovine serum (FBS) at 37 °C in a humidified incubator containing 5% CO_2_.

### Establishment of DOX-resistant gastric cancer cell line

The DOX concentration gradient progressive increase induction method was used to develop DOX-resistant phenotype SGC7901 cell line. SGC7901 cells at a concentration of 1 × 10^5^/ml were inoculated in DOX-free culture medium for 24 h until they got in the logarithmic phase. Then the culture medium was replaced with that containing low concentration (0.125 mg/L) DOX (Nanfang Hospital, Guangzhou, China, dissolved in RPMI 1640 medium) for 48 h. Then the culture medium containing drugs and dead cells was discarded. Cells were collected and re-inoculated in DOX-free culture medium to get recovered before next DOX treatment. After cells got adjusted to present DOX treatment, we increased the concentration of DOX and repeated the DOX and DOX-free treatment in turn until cells survived well and developed resistance to 0.75 mg/L DOX treatment. We referred to this DOX-resistant SGC7901 cell line as SGC7901/DOX. Both DOX- sensitive SGC7901 and DOX-resistant SGC7901/DOX were cultured under the same conditions and in drug-free medium for 2 days before commencing the experiments.

### CCK-8 assay

Cell survival rates were estimated by the CCK-8 assay (keyGEN biotech, Jiangsu, China). Approximately 10^4^ cells were seeded in 96-well plates with 100 μl medium each well. After 24 h cultivation, different doses of DOX or/and RES were added respectively. Each well was incubated with 10 μg CCK-8 solution for 2 h away from light before measuring the absorbance at 450 nm by PerkinElmer’s EnSpire Multilabel Plate Reader.

### Cell scratch test

When the cells seeded in 6-well plate reached a confluent state, a single scratch was made using a sterile 10 μl pipette tip. The cells were then incubated with FBS-free culture medium (to exclude the potential effects of FBS on cell migration) alone or containing different concentrations of DOX or RES (SinaBestBio, Shanghai, China, dissolved in 10% DMSO (DMSO:RPMI 1640 medium = 1:9)). Images of the scratches were captured at 0, 24 and 48 h with Olympus IX71 inverted microscope at 100× magnification. The width of the scratch was analyzed using the Olympus CellSens Dimension software.

### Transwell migration assay

Transwell migration assay was performed using transwell inserts (BD bioscience, SanJose, CA) with a filter of 8 μm pore. Cells were left untreated or treated with DOX, RES or both for 48 h. 8 × 10^4^ cells in serum-free medium were seeded into the upper chamber of the insert and complete medium was added to the lower chamber. After 36 h incubation, the cells were fixed with methanol and stained with Giemsa. Then cells on the top surface of the membrane were wiped off, and cells on the lower surface were examined with Olympus DP72 microscope at 100× magnification. 4 random fields were photographed for counting purposes and the average number of migrated cells was used as a measure of migration capacity.

### Colony-forming assay

Cells were left untreated or treated with DOX, RES or both for 48 h. Then cells were trypsinized and dispensed into individual wells of six-well tissue culture dishes with a density of 300 cells per well. Following another 14 days in drug-free culture, cells were fixed and stained with Giemsa to visualize colonies. Experiments were performed in triplicate.

### Apoptosis analysis

To detect cell apoptosis, cells were incubated with DOX or RES at different concentrations for 24 h. About 1 × 10^5^ cells were harvested by EDTA-free trypsinization and washed twice with cold PBS. After being resuspended in 500 μL binding buffer, cells were stained with 5 μL Annexin-V-FITC and 5 μL PI (Keygen bioTECH, Jiangsu, China) in dark condition at room temperature for 15 min. Lastly, cell apoptosis was measured by FACSAria flow cytometer (BD, USA).

### Immunofluorescence

The cells seeded in co focal dishes were fixed with 4% paraformaldehyde and then kept stable in 0.2% Triton for 10 min to rupture the cell membranes. Following three PBS washing, non-specific antigen-binding sites were blocked by 2% BSA for 30 min. The cells were then incubated with anti- Vimentin, E-cadherin (Abcam, 1:200); anti-β-catenin (Proteintech, 1:50) overnight at 4 °C. After washing, cells were incubated with anti-rabbit antibody for 60 min and the nuclei were stained with DAPI for 2 min, which was washed with PBS later. Cells were kept from light before observed with a fluorescence microscope.

### Western blot analysis

Cells to be tested were homogenized in Lysis Buffer (containing 0.1% protease inhibitor, 0.5% 100 mM PMSF and 1% phosphatase inhibitor) and centrifuged at 12,000 rpm for 15 min at 4 °C. The supernatant was collected and the total protein content was determined by BCA assay. Equal proteins (40 μg/lane) were loaded on SDS-PAGE gels and then transferred to 0.22 μm PVDF membranes. Membranes were blocked with 5% fat-free milk (5% skimmed milk powder in PBST, 0.1% Tween in PBS) and incubated with primary antibodies overnight at 4 °C. After being washed by PBST for 5 × 5 min, membranes were incubated with secondary antibodies for 1 h at room temperature. Following washing, target bands were visualized using Tanon-5200 Image Analyzer.

Primary antibodies used included rabbit monoclonal antibodies anti- PTEN, Akt, phosphor-Akt, mTOR, phosphor-mTOR, TSC1, TSC2, p70S6K, β-catenin, cleaved caspase-3 and caspase-9 (1:500, Cell Signaling Technology, Danvers, MA); rabbit monoclonal antibodies anti- Vimentin, E-cadherin (1:500, Abcam, Cambridge, UK); mouse monoclonal antibody anti-GAPDH (1:500, Proteintech, Chicago, IL). Protein bands were quantified using the Tanon-5200 Image Analyzer. All bands were normalized to GAPDH and the fold changes were calculated through relative quantification to the control group.

### Nude mice xenograft model

Nude mice were purchased from Guangdong Medical Laboratory Animal Center (Guangzhou, China) and housed under controlled temperature (28 °C) and pathogen-free conditions at Guangdong Provincial Key Laboratory of Molecular Tumor Pathology. All procedures were performed aseptically in accordance with the institutional guidelines of Guangdong Province and approved by the Experimental Animal Ethics Committee of Southern Medical University. 1 × 10^7^ SGC7901/DOX cells were injected subcutaneously into the left inguinal region of each nude mouse when the mice got five weeks old. Two weeks later, mice were randomly divided into four groups (*n* = 5, 3 males, 2 females): Group 1 Control; Group 2 DOX (3 mg/kg); Group 3 RES (50 mg/kg); Group 4DOX (3 mg/kg) and RES (50 mg/kg). DOX was diluted in sodium chloride injection and given intraperitoneally (i.p.) while RES was dissolved in 10% DMSO and administered by gavage. Both drugs were given once a week for 4 consecutive weeks. The tumor volume (V) was determined by measuring the length (a) and width (b) with calipers every week and calculated using the formula: V (mm^3^) =1/2*ab^2^. At the end of the treatment period, mice were sacrificed, and tumors were collected for immunochemical studies.

### Immunohistochemistry

The tumor tissue was fixed with 4% formalin and paraffin embedded before cutting it into 3 μm thick sections. And the sections were routinely deparaffinized in xylene and rehydrated in an alcohol gradient. Next, tissue antigen was retrieved by heating in sodium citrate (pH 6.0) or Tris-EDTA solution (pH 8.0) for 10 min. Sections were then blocked with 3% H2O2 before incubation with anti- caspase-3 (Cell Signaling Technology, 1:200); anti- ki67 (Cell Signaling Technology, 1:400); anti- Vimentin (Abcam, 1:500); anti-PTEN (Proteintech, 1:200) at 4 °C overnight. After three washes in PBS, the sections were incubated with secondary antibody (ZSGB-BIO, Beijing, China) for 30 min at room temperature. DAB kit (ZSGB-BIO, Beijing, China) was sequentially applied to stain the slides. Following counter-staining in hematoxylin and mounting with neutral balsam, the tissue sections were observed using Olympus BX-UCB light-field microscope. 5 random fields at × 400 magnification were captured for intensity analysis.

We used Image-Pro Plus software for semi-quantitative Image analysis. Firstly, we opened the image in the software and calibrated the optical density. In Measure menu we clicked Count/Size and chose Manual to enter into Segmentation dialogue box where the area of interest was set through: hue, 0 ~ 30; saturation, 0 ~ 255; intensity, 0 ~ 255, then the image was converted to gray scale image. Subsequently, we returned to Count/Size window, clicked Count and the values were measured. The parameters included area sum and IOD. The mean optical density was calculated by IOD/area and designated as representative staining intensity [19, 20].

### Statistical analysis

Data were expressed as means ± SD of at least three independent experiments. Statistical evaluation of the data was performed by using the unpaired Student’s *t*-test and ANOVA followed by a post-hoc test. Differences were considered to be statistically significant for *p <* 0.05. Statistical analysis was performed by SPSS 19.0 software.

## Results

### The effects of DOX and RES on gastric cancer cells

**Fig. 1:**
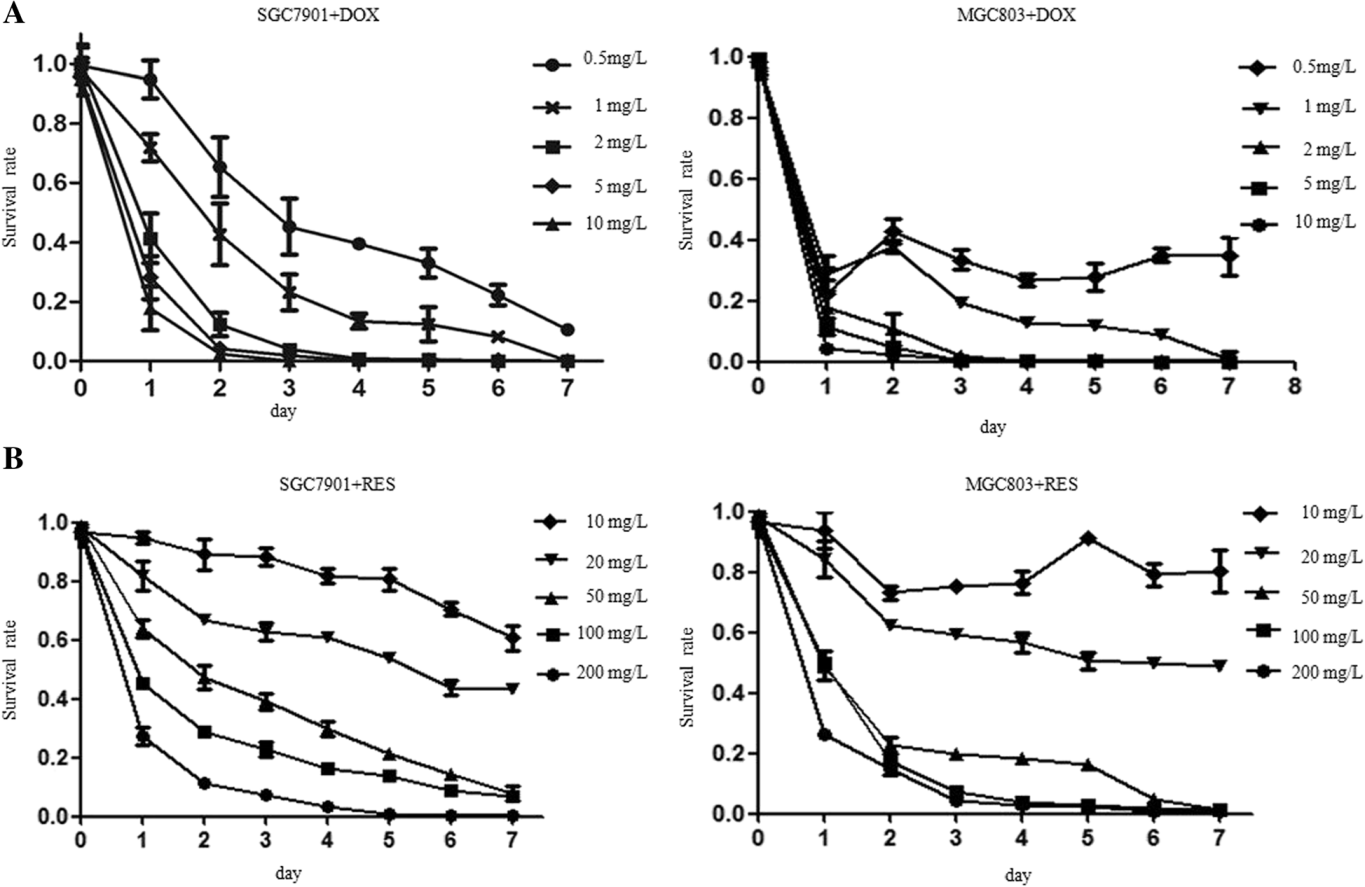
The effects of DOX and RES on gastric cancer cells. **a** CCK8 assay was used to detect the chemo-sensitiviy of SGC7901 and MGC803 cells to DOX treatment. Survival rate = (mean absorbance of experimental group/mean absorbance of control group) × 100%. The survival inhibition effect of doxorubicin demonstrated a dose (Factorial ANOVA, *p* < 0.001) and time-dependent (Factorial ANOVA, *p* < 0.001) manner, except that the difference between 10 mg/L and 5 mg/L (*p* = 0.910) and that between day 4 and day 5 (*p* = 0.300) were not significant. **b** The reveratrol concentration gradient ranges from 10 mg/L to 200 mg/L. The survival inhibition effect of resveratrol on SGC7901 cells demonstrated a dose (Factorial ANOVA, *p* < 0.001) and time-dependent (Factorial ANOVA, *p* < 0.001) manner. Also, the survival inhibition effect of resveratrol on MGC803 cells demonstrated a dose (Factorial ANOVA, *p* < 0.001) and time-dependent (Factorial ANOVA, *p* < 0.001) manner when resveratrol concentration was over 20 mg/L

We detected the chemo-sensibility of SGC7901 and MGC803 to DOX and RES treatment via CCK8 assay. The DOX concentration gradient ranges from 0.5 mg/L to 10 mg/L. Results showed SGC7901 cell survival was inhibited by DOX and the inhibition rate was increased with DOX treatment time and concentration. However, DOX did not suppress MGC803 cell survival in a dose and time-dependent way until its concentration was over 2 mg/L. Particularly, MGC803 survival rate could reach as high as 40% after 7-day treatment of 0.5 mg/L DOX (Fig. 1a). The RES concentration gradient ranges from 10 mg/L to 200 mg/L. Results showed that RES also significantly inhibited SGC7901 cell survival dose and time-dependently within the concentration range set. However, RES did not suppress MGC803 cell survival in a dose and time-dependent way until its concentration was over 20 mg/L (Fig. 1b). The 7-day survival of MGC803 maintained over 80% when treated with 10 mg/L RES and about 60% in 20 mg/L RES.

### Drug-resistant SGC7901/DOX cells were established and demonstrated enhancive migratory phenotype

Overall, DOX and RES demonstrated similar effects on the survival of both gastric cancer cell lines. But compared with MGC803, SGC7901 was more sensitive to both drugs, which made it a proper subject of our further study. The half maximal inhibitory concentration (IC50) of DOX in day2 for SGC7901 was 0.7635 mg/L (with 95% confidential interval 0.6765 to 0.8617 mg/L).

**Fig. 2:**
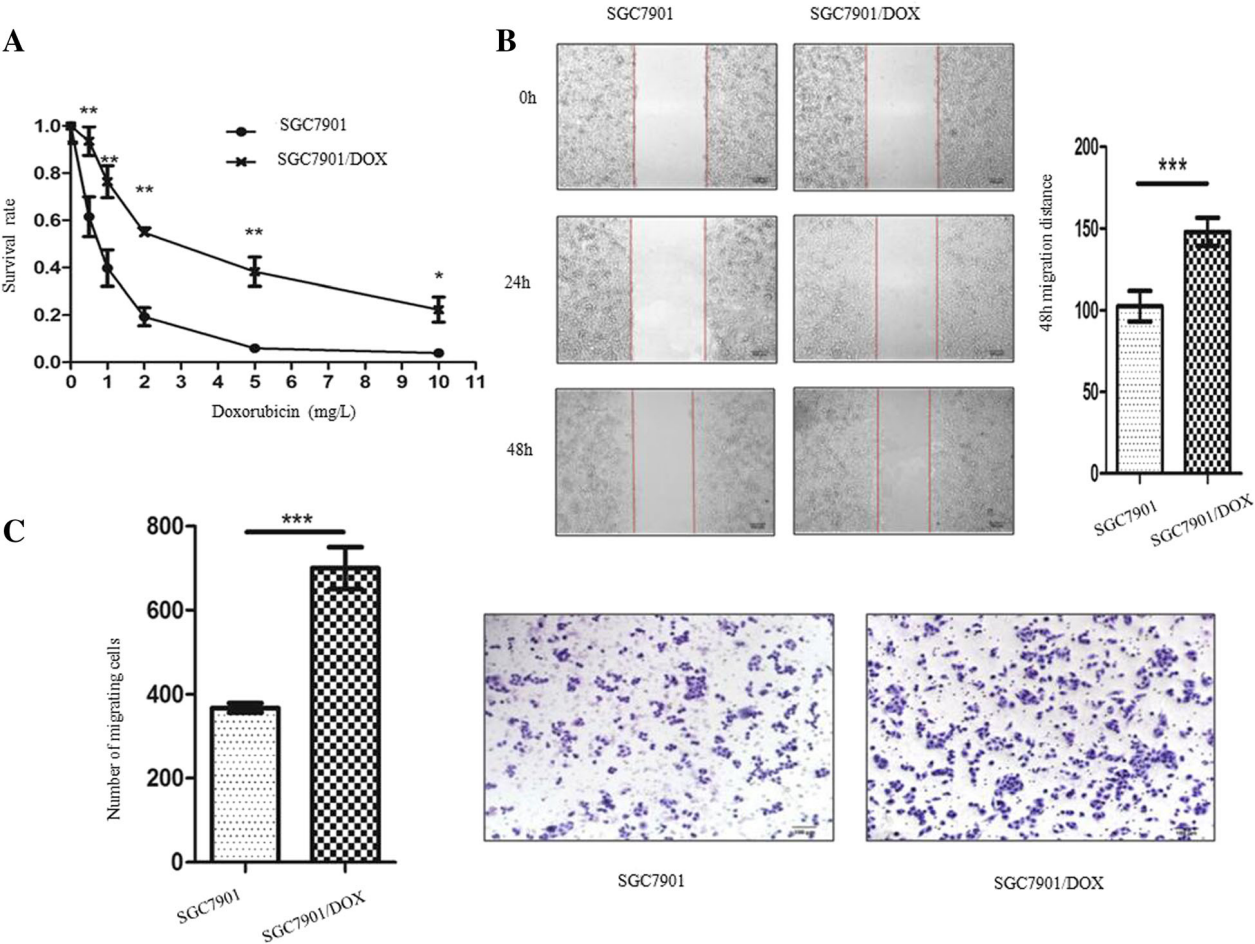
Drug-resistant SGC7901/DOX cells were established and demonstrated enhancive migratory phenotype. **a** CCK8 assay was used to evaluate the chemo-sensitiviy of SGC7901 cells and SGC7901/DOX cells to different concentrations of DOX after 48-h treatment (*n* = 4, **P < 0.001, *P < 0.01). **b** Cell scratch test was performed to measure the migration ability of SGC7901 and SGC7901/DOX cells. The Scale bar represents 100 μm. The data are represented as mean ± SD of four independent experiments (*n* = 4, *** *p* < 0.001). **c** Representative photographs of transwell migration assay and the number of transmembrane cells. The Scale bar represents 100 μm. Data represent as mean ± SD of four independent experiments (*n* = 4, *** *p* < 0.001)

DOX-resistant subclone cell line (SGC7901/DOX) was derived from SGC7901 cells exposed to stepwise increasing concentrations (0.125 mg/L to 0.75 mg/L) of DOX treatment. To verify the DOX-resistant difference between SGC7901 and SGC7901/DOX cells, we treated both cell lines with different concentrations of DOX for 2 days and then measured the half maximal inhibitory concentration (IC50). The IC50 of parental SGC7901 cell line was 0.7173 mg/L, and its 95% confidential interval (CI) was 0.6415 to 0.8019 mg/L while that of SGC7901/DOX cell line was 2.959 mg/L with a 95% CI ranging from 2.554 to 3.428 mg/L. The results showed that SGC7901/DOX cells were more DOX-resistant in comparison to its parental SGC7901 cells for its IC50 was 316% greater than that of SGC7901 (Fig. 2a).

Cell migration was analyzed by cell scratch test and transwell assay. Cell scratch test confirmed the migration potential of SGC7901/DOX (147.91 ± 4.32 μm) cells was 44.27% greater than that of SGC7901 cells (102.52 ± 4.72 μm, *p* < 0.001) (Fig. 2b). Transwell assay also reported SGC7901/DOX cells acquired enhancive cell migratory ability than parental SGC7901 cells, which was evidenced by increased number of trans-membrane migrating cells by 90.53% (700.40 ± 22.38 SGC7901/DOX cells compared with 367.60 ± 5.07 SGC7901 cells (*p* < 0.01) (Fig. 2c).

### SGC7901/DOX cells underwent EMT associated with Akt activation

**Fig. 3:**
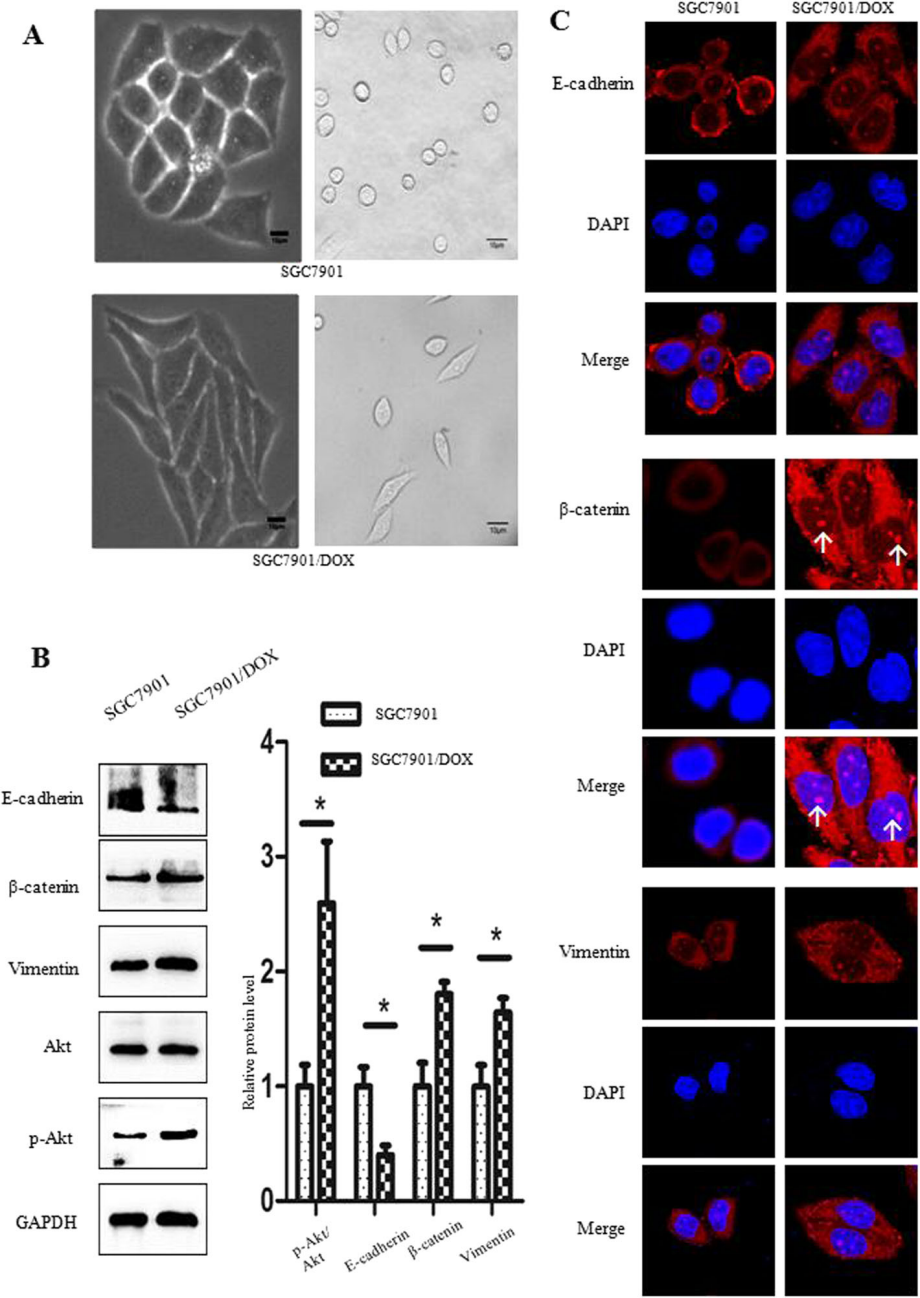
SGC7901/DOX cells underwent EMT mediated by upregulation of Akt signaling pathway. **a** Images were captured by Olympus IX71 inverted microscope system. Morphological observation showed morphological variance between SGC7901/DOX and SGC7901 cells. The Scale bar represents 10 μm. **b** SGC7901 and SGC7901/DOX cells were left DOX-untreated for 48 h for Western blot assay, measeuring EMT-related proteins and Akt expression. Bar diagram shows the relative expressions of proteins normalized to GAPDH. Data are represented as mean ± SD of three independent experiments (*n* = 3, *** *p* < 0.001; ** *p* < 0.01; * *p* < 0.05: NS means not significant, *p* > 0.05). **c** Immunofluorescence assay was used to detect EMT-associated proteins in SGC7901/DOX cells. E-cadherin, β-catenin, and vimentin were stained red and nuclei stained with DAPI were blue. The white arrow indicates β-catenin nuclear tranlocation. Images were captured at 1800× magnification

EMT has been illustrated as a critical regulator in cell migration and drug resistance. Morphological observation showed that the parental SGC7901 cells were generally round or oval, but most of SGC7901/DOX cells transformed into spindle shape (Fig. 3a). The expression of EMT associated markers was measured by western blot. SGC7901/DOX cells were found to undergo EMT process for it expressed higher mesenchymal cell markers including β-catenin and vimentin while losing epithelial cell adhesion molecule such as E-cadherin. In addition, Akt was activated in SGC7901/DOX cells (Fig. 3b). EMT markers were also verified through immunofluorescent assay. Long-term DOX incubation decreased E-cadherin cytomembrane expression whereas increased vimentin expression in cytoplasm and enhanced β-catenin nuclear tranlocation (Fig. 3c).

### RES alleviated the aggressive phenotypes of SGC7901/DOX cells

**Fig. 4:**
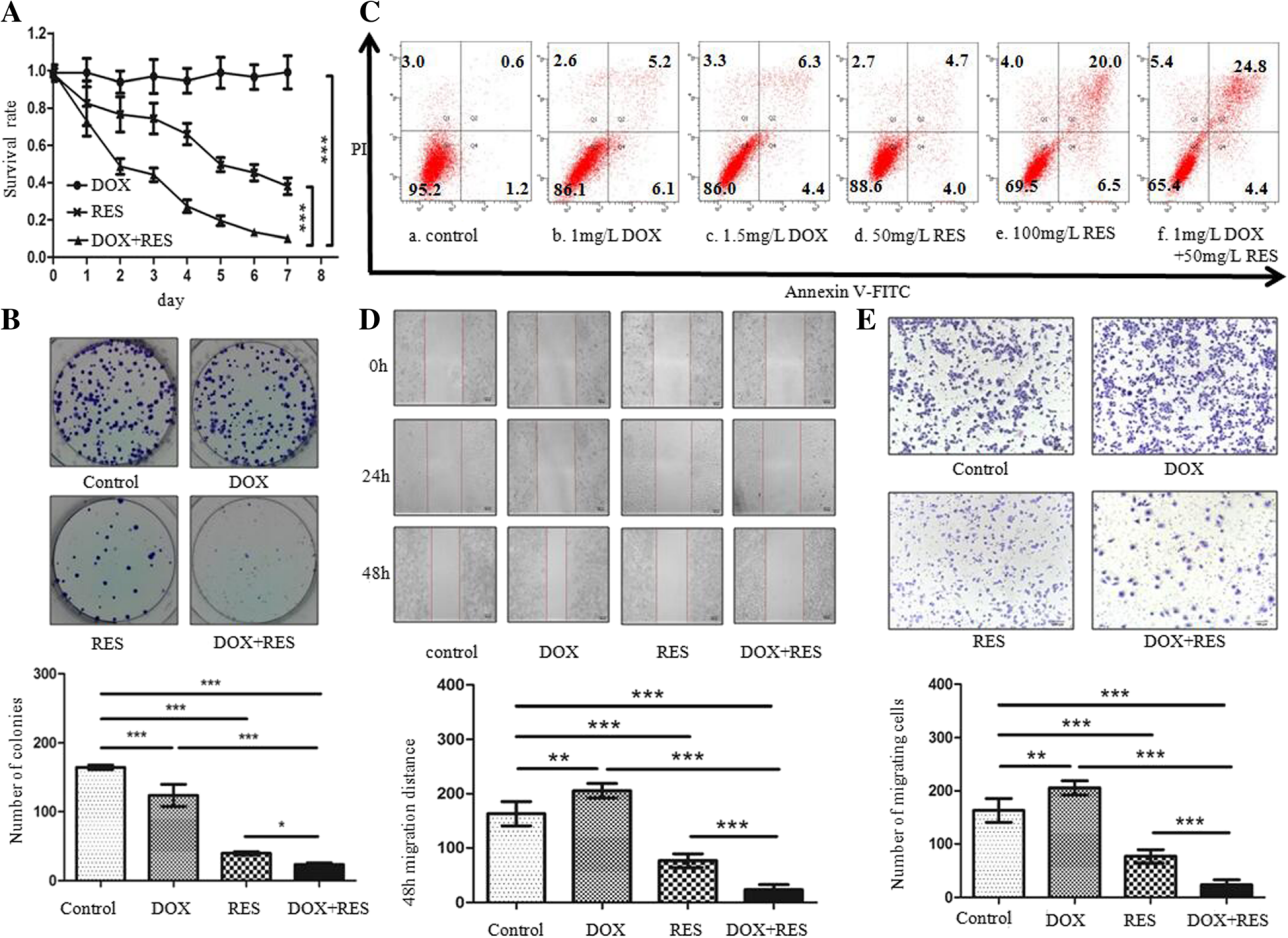
RES synergized DOX effect on cell proliferation, colony formation and apoptosis and resvered DOX-induced cell migration in SGC7901/DOX cells. **a** CCK8 was used to detect the cytotoxicity of DOX, RES or both. SGC7901/DOX cells were left untreated or treated with 0.75 mg/L DOX, 50 mg/L RES or both for 7 days. Each data point represents a mean value of four experiments and the error bars indicate the standard deviation (*T* test, vs. DOX + RES, *, *p* < 0.01; **, *p* < 0.001, *n* = 4). **b** Representative pictures of Colony-forming assay and number of cell colonies. After 48 h’ exposure to DOX or RES or both, the colony forming abilitiy of SGC7901/DOX cells was tested. (*n* = 3, *** *p* < 0.001; *, *p* < 0.05). **c** SGC7901/DOX cells were treated with 1 mg/L DOX, 1.5 mg/L DOX, 50 mg/L RES, 100 mg/L RES or 0.75 mg/L DOX combined with 50 mg/L RES respectively for 48 h. Annexin V-FITC/PI dual staining apoptosis analysis was performed. The proportions of cells in each quadrant are marked on the figures. **d** The migration distance was meaured to analysed the migration ability of SGC7901/DOX cells which were left untreated or treated with 1 mg/L DOX, 50 mg/L RES or both for 48 h. The Scale bar represents 100 μm. The migration distance of each group was measured, with 162.89 ± 11.20 μm, 205.11 ± 6.79 μm, 76.34 ± 6.16 μm, 24.36 ± 4.83 μm for control, DOX, RES and DOX + RES group. (*n* = 4, *** *p* < 0.001;**p < 0.01). **e** SGC7901/DOX cells were left untreated or treated with 1 mg/L DOX, 50 mg/L RES or 1 mg/L DOX simultaneously combined with 50 mg/l RES for 48 h. Then cells were subjected to transwell migration assay. The Scale bar represents 100 μm. The numbers of cells of the control, DOX, RES and DOX + RES groups were 700.40 ± 50.03, 922.00 ± 53.25, 271.60 ± 20.07 and 116.00 ± 6.50 respectively (*n* = 4, *** *p* < 0.001;**p < 0.01)

To evaluate the cytotoxic effect of RES on SGC7901/DOX cells, cells were treated with 0.75 mg/L DOX or 50 mg/L RES, either alone or in combination. CCK8 assay results showed DOX (0.75 mg/L) had no significant effect on the survival of SGC7901/DOX cells, which indicated that SGC7901/DOX cells maintained DOX-resistant feature as previously described. As for the RES group, RES treatment displayed a time-dependent inhibitory effect on SGC7901/DOX cell survival. Notably, when DOX was combined with RES, the survival rate of SGC7901/DOX cells was minimized (Fig. 4a).

Furthermore, we analyzed whether the combined treatment could suppress the colony-forming ability of SGC7901/DOX cells. DOX and RES treatment decreased the number of SGC7901/DOX cell colonies by only 33.95 and 76.54% respectively. When DOX and RES were combined, the colony forming ability of SGC7901/DOX cells reached the lowest point, much lower than either treatment alone (Fig. 4b).

Also, the effect of RES and/or DOX on cell apoptosis was measured. DOX treatment alone could induce SGC7901/DOX cell apoptosis, but it was not prominent at low concentrations (1 mg/L and 1.5 mg/L). RES alone triggered cell apoptosis in a concentration-dependent manner, with 8.7% apoptotic cells resulting in 50 mg/L treatment and then 26.5% in 100 mg/L. Altogether, 1 mg/L DOX and 50 mg/L RES combined treatment synergistically promoted cell apoptosis proportion up to 29.2% (Fig. 4c). The results revealed that RES increased the DOX-sensibility of SGC7901/DOX cells by synergizing DOX’s function in inhibiting cell proliferation and promoting cell apoptosis.

The effect of RES on SGC7901/DOX cell migration was also investigated. To our surprise, cell scratch test suggested that DOX treatment alone promoted the migration of SGC7901/DOX cells by 25.9% whereas RES decreased that of SGC7901/DOX by 53.13%. Notably, combination of DOX and RES achieved a remarkably stronger migration-inhibitory effect than RES or DOX treatment alone (Fig. 4d). According to transwell assay results, after 48 h DOX treatment, the number of trans-membrane SGC7901/DOX cells was increased by 31.64%. However, RES, whether alone or combined with DOX, drastically decreased migrating cell number (Fig. 4e). Therefore, RES effectively prohibited SGC7901/DOX cells migration and what’s more, it significantly antagonized and reversed SGC7901/DOX cells migration when DOX was present.

### RES combined with DOX could reverse EMT

**Fig. 5:**
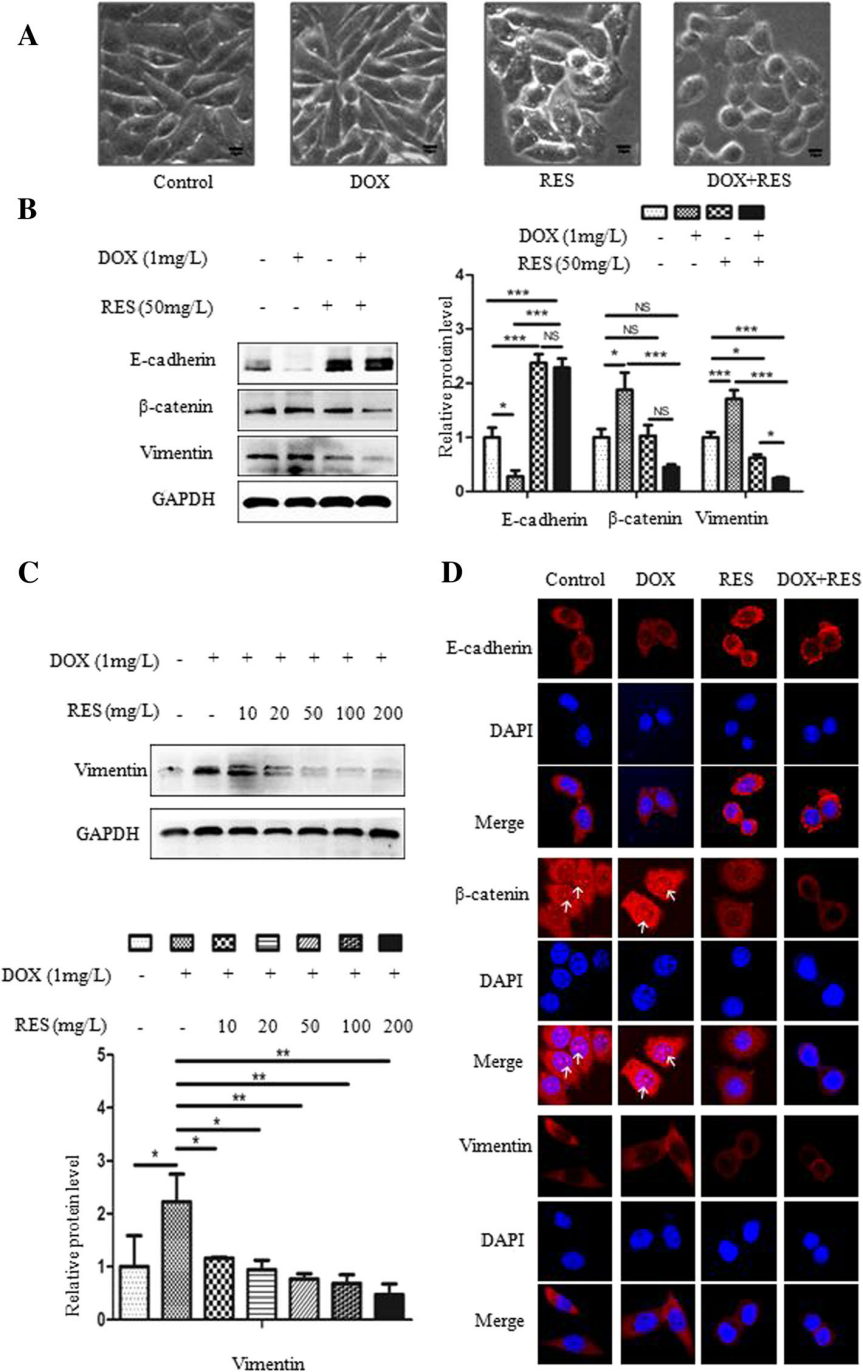
Effect of RES on DOX-induced EMT. SGC7901/DOX cells were untreated or treated with 1 mg/l DOX, 50 mg/l RES or 1 mg/l DOX simultaneously combined with 50 mg/l RES for 48 h. **a** Representative images of treated cells were shown. Scale bar represents 10 μm. **b**, **c** After 48 h treatment, cells were harvested for Western blot analysis to detect whether DOX and RES affect the expression of EMT-related proteins. Bar diagram shows the relative expressions of proteins normalized to GAPDH. Data are represented as mean ± SD of three independent (*n* = 3, *** *p* < 0.001; ** *p* < 0.01; * *p* < 0.05: NS means not significant, *p* > 0.05). **d** Immunofluorescence assay was used to detect EMT-related proteins. Vimentin, E-cadherin and β-catenin were stained red and nuclei stained with DAPI were blue. The white arrow indicates β-catenin nuclear tranlocation. Images were captured at 1800× magnification

To verify the migration-inhibitory effect of RES was associated with the mitigation or even reversion of EMT, morphological changes among SGC7901/DOX cells treated with DOX, RES or both for 48 h were captured. SGC7901/DOX cells, no matter treated with DOX or not, demonstrated spindle or shuttle shapes, but they turned round or oval when RES was present (Fig. 5a).

Then the expression of EMT-related proteins was detected by western blot and immunofluorescent assay for further verification. Similar to our previous findings in long-term DOX treated SGC7901/DOX cells, 48-h DOX treatment on SGC7901/DOX cells enhanced the expression of mesenchymal cell markers such as vimentin and β-catenin, and reduced that of E-cadherin, an epithelial cell adhesion molecule. RES treatment, whether combined with DOX or not, decreased the expression of vimentin and increased E-cadherin expression dominantly, but had not significantly changed that of β-catenin when treated alone. However, when DOX was present, RES significantly antagonized DOX-induced upregulation of β-catenin. And compared with either treatment alone, vimentin expression was significantly downregulated in DOX-RES combined treatment cells (Fig. 5b), which was consistent with its cell migration suppressing effect. Moreover, the downregulation of vimentin expression on combined treatment cells was intensified as RES concentration increased (Fig. 5c). Similar cellular changes of E-cadherin, β-catenin and vimentin expression were verified in immunofluorescent assay (Fig. 5d). These studies showed that RES antagonized DOX-induced EMT through the downregulation of vimentin and β-catenin, and upregulation of E-cadherin.

### RES modulated PTEN/Akt signaling pathway in SGC7901/DOX cells

**Fig. 6:**
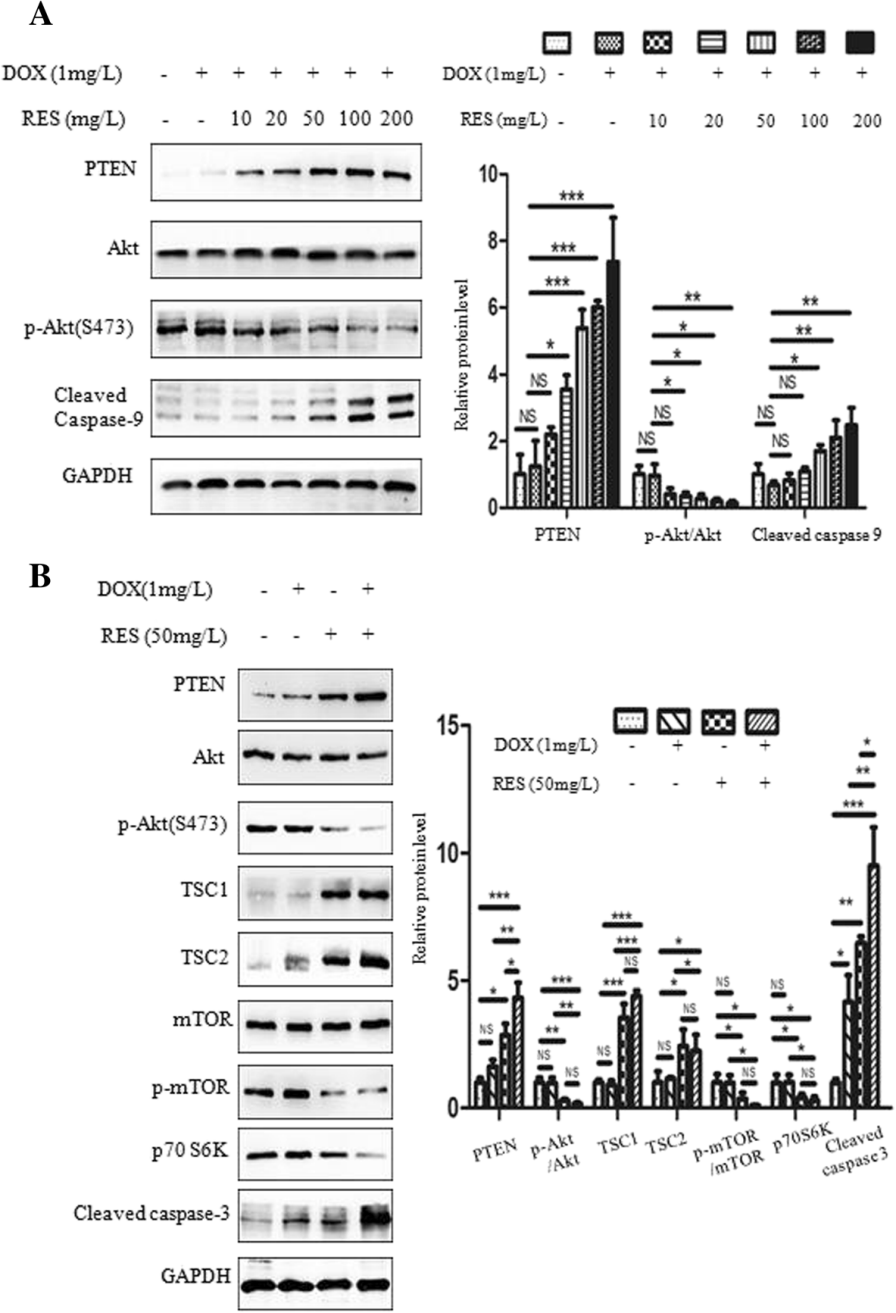
Effect of RES on PTEN/Akt signaling pathway in SGC7901/DOX cells. **a**, **b** Following 48 h treatment, SGC7901/DOX cells were harvested for Western blot analysis to evaluate the effect of DOX and RES on PTEN/Akt signaling expression. Bar diagram shows the relative expressions of proteins normalized to GAPDH. Data are represented as mean ± SD of three independent (*n* = 3, *** *p* < 0.001; ** *p* < 0.01; * *p* < 0.05: NS means not significant, *p* > 0.05)

To gain insight into the molecular mechanism through which RES reversed EMT, the expression of PTEN/Akt signaling pathway was analyzed by western blot. Results indicated that 1 mg/L DOX treatment alone did not affect PTEN expression significantly. Along with the combination of 1 mg/L DOX, however, RES significantly activated PTEN expression and prohibited Akt phosphorylation as its concentration increased from 20 mg/L to 200 mg/L. Moreover, the expression of cleaved caspase-9 was also enhanced in a RES-dependent way when RES concentration ranged from 50 mg/L to 200 mg/L (Fig. 6a). To detect whether RES modulates PTEN/Akt signaling pathway as a whole, we treated SGC7901/DOX cells with 1 mg/L DOX, 50 mg/L RES or both. Consistant with previous observation, in 50 mg/L RES treatment group, we saw an significant increase in the level of PTEN, TSC1, TSC2 and cleaved caspase-3 and a reduction in p-Akt, p-mTOR and p70 S6K. Notably, when RES was combined with DOX, PTEN upregulation and caspase-3 cleavage were strengthened in comparison to RES treatment alone (Fig. 6b). This study verified that RES suppressed Akt signaling pathway by upregulating PTEN.

### RES and DOX combination synergistically delayed tumor growth through reverse of EMT and promotion of apoptosis by activating PTEN in vivo

**Fig. 7:**
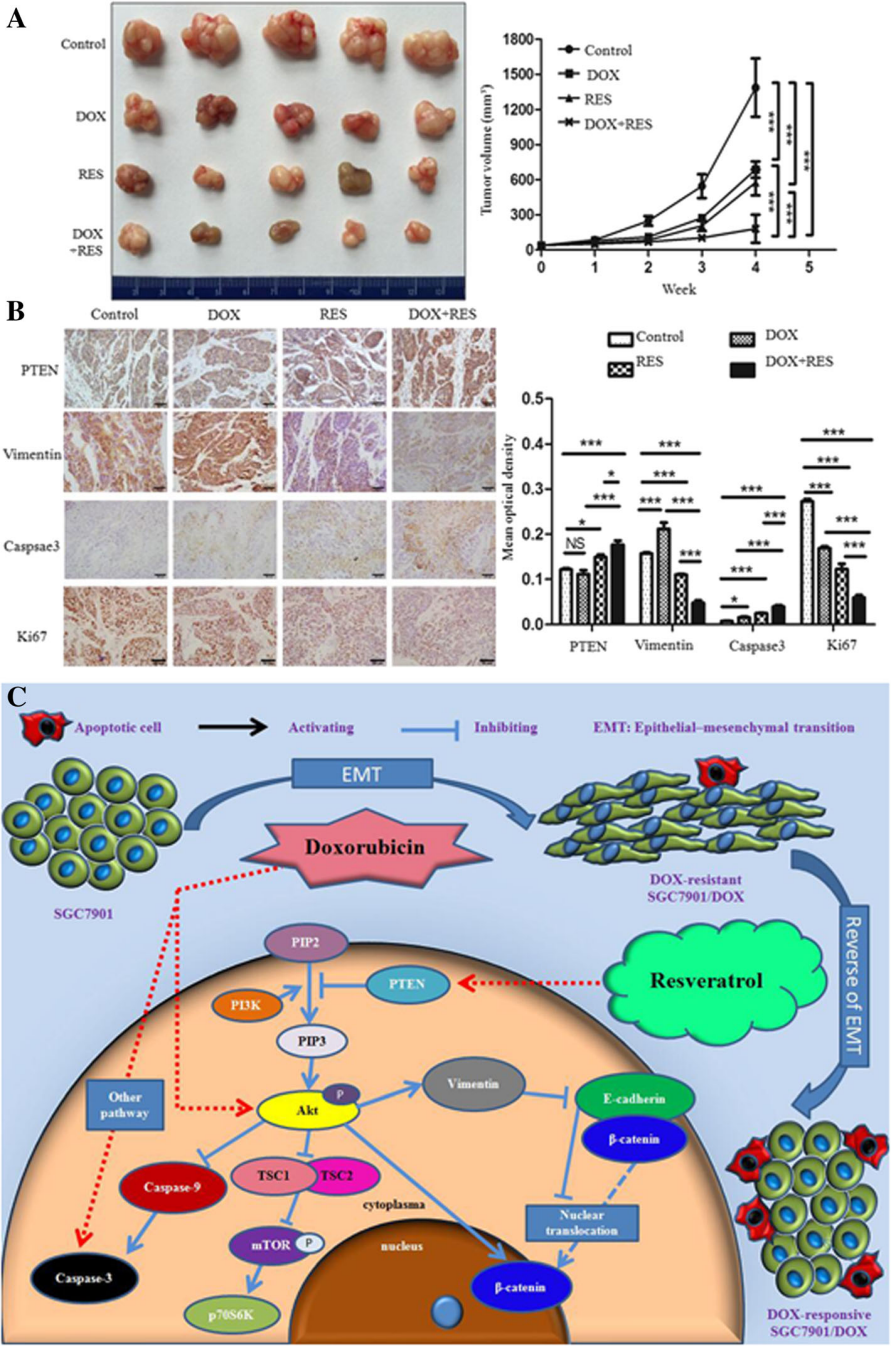
Effect of DOX and RES on nude mice burdened with SGC7901/DOX xenograpfts*.* **a** Nude mice inoculated subcutaneously with SGC7901/DOX cells were treated with DOX, RES or both for 4 weeks. At the end of the treatment period, mice were sacrificed and tumor specimens harvested. The tumor volume (V) was determined by measuring the length (a) and width (b) with calipers every week and calculated using the formula: V (mm^3^) =1/2ab^2^. Mean tumor volume for each treatment group is indicated. Results are represented as the mean ± SD of 5 mice per group (*n* = 5,***p < 0.001). **b** Tumor specimens were subjected to immunohistochemical staining for PTEN,Ki67, caspase3 and vimentin. Images were captured at 400× magnification. The Scale bar represents 50 μm. Mean density of each group (Integrated optical density/Area) was presented (*n* = 5, *** *p* < 0.001; * *p* < 0.05;NS means not significant, *p* > 0.05). **c** A hypothetical model illustrating that RES reverses DOX-induced EMT and DOX-resistance by modulating PTEN/Akt signaling pathway. This picture was designed by J.X. and Q.Z

To evaluate the effect of DOX and RES on tumor growth, nude mice bearing subcutaneous SGC7901/DOX xenograpfts (with mean tumor volume 36.46 ± 13.82 mm^3^) were given DOX, RES or both for 4 consecutive weeks. Administration of DOX (3 mg/kg) reduced the tumor volume by 50.44%, from 1387.10 ± 248.02 mm^3^ of Control to 687.45 ± 69.69 mm^3^ (*p* < 0.001), and RES (50 mg/kg) reduced the tumor volume by 58.42% to 576.80 ± 110.64 mm^3^ (*p* < 0.001). And likewise, the novel regimen of combinatorial treatment achieved a synergistic tumor-inhibitory effect, minimizing the tumor volume by 86.97% to 180.75 ± 119.38 mm^3^ (*p* < 0.001), which was 73.71% (*p* < 0.001) and 68.66% (*p* < 0.001) lower than DOX and RES treatment respectively (Fig. 7a).

To further validate the molecular basis, we then performed immunohischemistry on the tumor xenografts to detect the level of proliferation marker Ki67, EMT-related vimentin, apoptosis-related caspase 3 and the Akt signaling inhibitor PTEN in vivo*.* As expected, DOX alone did not affect PTEN expression, but significantly increased the expression of vimentin and caspase-3 and reduced Ki67 expression. Compared with control group, RES alone evidently enhanced PTEN and caspase-3 expressions while reducing Ki67 and vimentin expressions. Moreover, when combined with DOX, RES achieved a much more dramatic enhancive effect on PTEN and caspase-3, and displayed a more remarkable inhibitory effect on vimentin and Ki67 (Fig. 7b).

## Discussion

Chemotherapy, which provides palliation of symptoms and improves survival and life quality, is the most effective treatment for patients with inoperable cancers. However, conventional DOX-based chemotherapy regimen has been criticized for a series of negative effects, including the development of drug resistance and the occurrence of Epithelial–mesenchymal transition (EMT) [21, 22].

EMT not only enhances the metastatic potentials of cancer but also participates in the development of chemo-resistance [23, 24]. EMT is the physiological or pathological conversion of epithelial cells to mesenchymal cells, in which cells undergo phenotypic changes including the loss of cell polarity and cell-cell adhesion, the acquisition of migratory and invasive properties, which are highly responsible for carcinoma progression. The EMT-induced stemness endows cancer cells with the ability to overexpress chemo-resistance related genes, leading to multiple drug resistance in cancer treatment [25]. A previous study detected DOX-induced EMT in BGC823 gastric cancer cells. Inhibition of β-catenin signaling could suppress DOX-induced EMT and cell migration [22]. Suppression of EMT through selective inhibition of β-catenin signaling could restore sensitivity to HER-2 targeted lapatinib in HER-2 positive gastric cancer cells SNU216 cells [26]. Very recently, an EMT lineage-tracing system was established to monitor reversible and transient EMT process in mice. Upon treatment with cancer chemotherapy drug cyclophosphamide, EMT cells were detected in the primary tumor and showed chemo-resistance owing to reduced proliferation, apoptotic tolerance and increased expression of chemoresistance-related genes. Theses EMT cells also contributed to recurrent lung metastasis formation after chemotherapy. These data suggested that EMT plays an important role in cancer drug resistance and contributes to metastasis after chemotherapy treatment [27]. In our study, we generated SGC7901/DOX cell line by long-term and incremental DOX treatment, which was characterized by the acquisition of drug resistance and enhancive migration (Fig. 2). And meanwhile, SGC7901/DOX cells displayed an apparent EMT potential for they were transformed into spindle-like shape, and expressed high level of mesenchymal cell markers including β-catenin and vimentin while losing epithelial cell adhesion molecule such as E-cadherin (Fig. 3).

EMT-mediated therapeutic resistance in solid tumors is regulated by many canonical signaling pathways, among which PI3K/Akt is of high interest. [28, 29] PI3K phosphorylates PIP2 into PIP3, which then phosphorylates Akt in turn. Akt gets activated by phosphorylation of its Ser473 residue and stimulates the mTOR complex 1 (mTORC1) by phosphorylating tuberous sclerosis complex2 (TSC2) and subsequently inhibiting TSC1/2 complex formation, which is a negative regulator of mTORC1. Further, the mTORC1 complex acts on RHEB (Ras homolog enriched in brain) to phosphorylate mTOR at Ser2448 and thus resulting in mTOR activation. Then mTOR regulates protein translation and cell growth by phosphorylating ribosomal S6 kinase (p70S6K) [30]. It has been reported that mTOR inhibitors decrease p70S6K and reverse resistance to DOX [31]. Another important substrate of Akt that initiates the mitochondrial apoptotic pathway is caspase-9, which is inactivated by Akt through the phosphorylation at Ser196 [32]. Caspase-9 inactivation then leads to the downregulation of caspase-3 and suppression of caspase-dependent apoptosis [33]. As other study shows, Akt activation induces cancer cell invasiveness partially through interaction with vimentin. The binding of Akt to vimentin enhances the ability of vimentin to induce motility and invasion while protecting vimentin from caspase-induced proteolysis [34]. Vimentin promotes cell invasiveness via the regulation of E-cadherin/β-catenin complex. [35] Compared with E-cadherin and β-catenin, vimentin seemed to be a more specific and significant determinant for cancer metastasis [36, 37]. Moreover, Akt triggers DOX-induced EMT through Akt/GSK-3β/β-catenin pathway. Nuclear translocation of β-catenin not only regulates EMT-associated proteins, which are responsible for cell migration, but also induces transcriptional upregulation of ZEB1, which in turn regulated DNA damage repair and DOX-resistance [38]. Recently, the promotion of Akt phosphorylation could facilitate EMT, resulting in tumor formation and invasion in AGS and MKN45 gastric cancer cell lines [39]. Given that Akt was activated in SGC7901/DOX cells, we are convinced that the upregulation of Akt signaling pathway best illustrates DOX-induced EMT, cell migration and drug resistance mentioned above. Even though activation of Akt signaling pathway was maintained by DOX treatment in SGC7901/DOX cells, caspase-3 mediated apoptosis was promoted by DOX in spite of caspase-9 inactivation by Akt. Therefore, DOX could induce SGC7901/DOX cell apoptosis in an Akt-independent way.

To alleviate the adverse effects of DOX, we took note of RES, a well-known phytochemical with diverse health benefits, as a possible supplement for DOX treatment. RES demonstrated its valid potentials as a chemosensitizer for DOX in breast cancer cells [40]. In gastric cancer MGC803 cells, RES was able to induce cell cycle arrest by targeting PTEN. As a negative regulator of PI3K/Akt pathway which dephosphorylates PIP3, PTEN has been reported to reverse EMT-mediated drug resistance [41]. Consistent with our data, an early study revealed RES is able to suppress cell invasion and the expression of Snail and N-cadherin and increase that of E-cadherin, which suggested that RES has an EMT-inhibitory effect in SGC7901 cells [42]. We discovered that PTEN expression was enhanced by RES dominantly and dose-dependently in DOX-resistant SGC7901/DOX gastric cancer cells. Consequently, PI3K/Akt pathway was down-regulated, which activated caspase-3 dependent apoptosis, suppressed colony formation, and more importantly, inhibited EMT, leading to the suppression of cell migration and the reverse of DOX-resistance of SGC7901/DOX cells. Similar effects were also confirmed in SGC7901/DOX in vivo xenograpft tumor model.

## Conclusions

Our study revealed that Akt aberrant activation-mediated EMT is the key to the acquisition of DOX-resistance in SGC7901 gastric cancer cells during long-term DOX treatment. RES could attenuate the adverse effects of DOX by activating PTEN and subsequently modulating Akt pathway activity, thus further promoting apoptosis and reversing EMT process. In conclusion, RES serves as a novel solution which can not only synergize DOX in inhibiting the tumor growth, but also reverse the DOX-resistance and prevent cell migration via the suppression of EMT in gastric cancer by modulating PTEN/Akt signaling pathway. The DOX-RES combined treatment provides a promising future for gastric cancer patients with metastasis and drug resistance tendency.

## Abbreviations

ANOVA: Analysis of variance
CCK8: Cell counting kit-8
DOX: Doxorubicin
EMT: Epithelial-mesenchymal transition
mTOR: Mammalian target of rapamycin
PTEN: Phosphatase and tensin homolog deleted on chromosome ten
RES: Resveratrol
