# Correction: Resveratrol reverses Doxorubicin resistance by inhibiting epithelial-mesenchymal transition (EMT) through modulating PTEN/ Akt signaling pathway in gastric cancer

**DOI:** 10.1186/s13046-023-02593-5

**Published:** 2023-01-17

**Authors:** Jiahui Xu, Deying Liu, Huilin Niu, Guifang Zhu, Yangwei Xu, Danli Ye, Jian Li, Qingling Zhang

**Affiliations:** 1 Nanfang Hospital/First Clinical Medical School, Southern Medical University, Guangzhou, 510515 China; 2 Department of Pathology, School of Basic Medical Sciences, Southern Medical University, Guangzhou, 510515 China; 3 Department of Pathology, Nanfang Hospital, Southern Medical University, Guangzhou, 510515 China

**Correction: J Exp Clin Cancer Res 36, 19 (2017)**

**https://doi.org/10.1186/s13046-016-0487-8**

Following the publication of the original article [1], author identified an error in Figure 4, specifically:

- Figure 4d – migration distance of DOX treated 24h was pasted by using DOX treated 0h

The correct figure is given below.

**Fig. 4:**
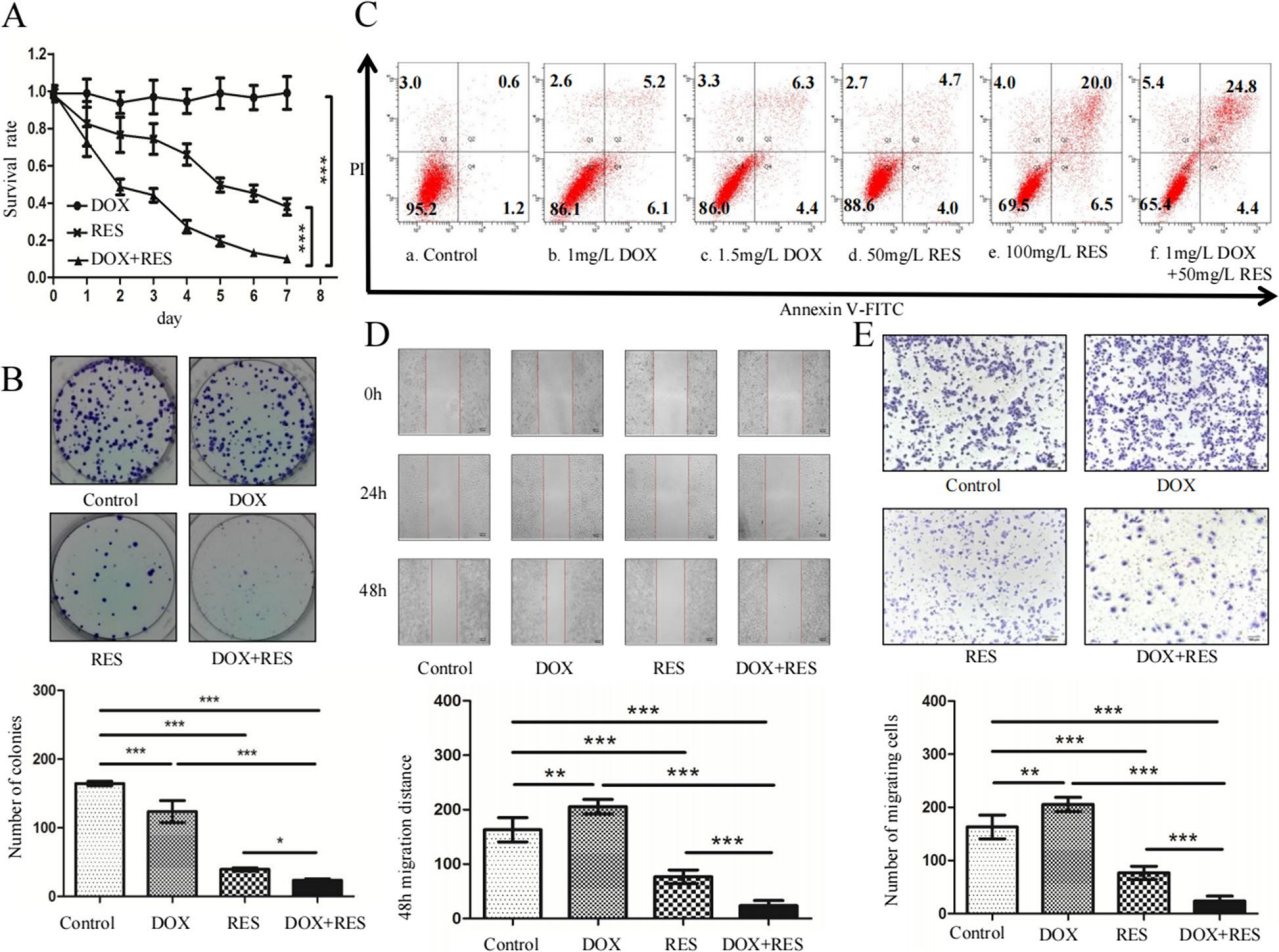
RES synergized DOX effect on cell proliferation, colony formation and apoptosis and resvered DOX-induced cell migration in SGC7901/ DOX cells. **a** CCK8 was used to detect the cytotoxicity of DOX, RES or both. SGC7901/DOX cells were left untreated or treated with 0.75 mg/L DOX, 50 mg/L RES or both for 7 days. Each data point represents a mean value of four experiments and the error bars indicate the standard deviation (*T* test, vs. DOX + RES, *, *p* < 0.01; **, *p* < 0.001, *n* = 4). **b** Representative pictures of Colony-forming assay and number of cell colonies. After 48 h’ exposure to DOX or RES or both, the colony forming abilitiy of SGC7901/DOX cells was tested. (*n* = 3, *** *p* < 0.001; *, *p* < 0.05). **c** SGC7901/ DOX cells were treated with 1 mg/L DOX, 1.5 mg/L DOX, 50 mg/L RES, 100 mg/L RES or 0.75 mg/L DOX combined with 50 mg/L RES respectively for 48 h. Annexin V-FITC/PI dual staining apoptosis analysis was performed. The proportions of cells in each quadrant are marked on the figures. **d** The migration distance was meaured to analysed the migration ability of SGC7901/DOX cells which were left untreated or treated with 1 mg/L DOX, 50 mg/L RES or both for 48 h. The Scale bar represents 100 μm. The migration distance of each group was measured, with 162.89 ± 11.20 μm, 205.11 ± 6.79 μm, 76.34 ± 6.16 μm, 24.36 ± 4.83 μm for control, DOX, RES and DOX + RES group. (*n* = 4, *** *p* < 0.001;**p < 0.01). **e** SGC7901/ DOX cells were left untreated or treated with 1 mg/L DOX, 50 mg/L RES or 1 mg/L DOX simultaneously combined with 50 mg/l RES for 48 h. Then cells were subjected to transwell migration assay. The Scale bar represents 100 μm. The numbers of cells of the control, DOX, RES and DOX + RES groups were 700.40 ± 50.03, 922.00 ± 53.25, 271.60 ± 20.07 and 116.00 ± 6.50 respectively (*n* = 4, *** *p* < 0.001;**p < 0.01)

## References

[1] Xu J, Liu D, Niu H (2017). Resveratrol reverses Doxorubicin resistance by inhibiting epithelial-mesenchymal transition (EMT) through modulating PTEN/Akt signaling pathway in gastric cancer. J Exp Clin Cancer Res.

